# Decoding the role of cancer-associated fibroblasts in cisplatin resistance of head and neck squamous cell carcinoma

**DOI:** 10.1016/j.isci.2026.117696

**Published:** 2026-09-22

**Authors:** Dongmei Liu, Dilong Yu, Mengyu Chen, Tian Dai, Xingnuo Li, Dan Su, Juyan Zheng, Yiwen Zhang

**Affiliations:** 1 Department of Pharmacology, College of Pharmaceutical Sciences, Zhejiang University of Technology, Hangzhou 310014, China; 2 Center for Clinical Pharmacy, Cancer Center, Department of Pharmacy, Zhejiang Provincial People's Hospital (Affiliated People's Hospital), Hangzhou Medical College, Hangzhou 310014, China; 3 Zhejiang Provincial Clinical Research Center for Malignant Tumor, Hangzhou 310014, China; 4 Zhejiang Key Laboratory of Precision Medicine Research on Head & Neck Cancer, Hangzhou 310014, China; 5 Department of Clinical Medicine, Hangzhou Medical College, Hangzhou 310014, China; 6 Department of Emergency Medicine, Huashan Hospital, Fudan University, Shanghai, People's Republic of China

**Keywords:** cisplatin resistance, cancer-associated fibroblasts, head and neck squamous cell carcinoma, tumor microenvironment, chemotherapy resistance

## Abstract

Cisplatin remains the primary chemotherapy drug for the treatment of head and neck squamous cell carcinoma (HNSCC). However, acquired resistance significantly limits its clinical application, contributing to treatment failure and a poor patient prognosis. Cancer-associated fibroblasts (CAFs) in the tumor microenvironment influence HNSCC sensitivity to cisplatin by modulating tumor cell responsiveness to treatment via paracrine signaling. CAFs impede deep penetration into tumors by remodeling the extracellular matrix (ECM). Various subtypes of cisplatin-resistant CAFs have been identified in HNSCC. Potential compounds targeting these subtypes could enhance cisplatin sensitivity. Simulation of the dynamic tumor microenvironment remains limited in research, hindering clinical translation. Bioinformatics has shifted research into cisplatin resistance in HNSCC. Single-cell sequencing and spatiotemporal transcriptomics could analyze the molecular profiles of CAFs that promote drug resistance in greater depth, potentially facilitating the development of highly targeted drugs and effective combination therapy strategies to overcome cisplatin resistance in HNSCCs.

## Teaser

Oncology; Clinical pharmacy

## Introduction

Head and neck squamous cell carcinoma (HNSCC) arises from the epithelial tissues of the oral cavity, pharynx, and larynx and accounts for approximately 90% of all reported head and neck cancers (HNC), the seventh most common disease globally, and the ninth leading cause of cancer-related deaths.^1^ In 2022, more than 389,000 new cases and over 188,000 deaths were reported globally.

Surgery and chemotherapy remain central components of HNSCC treatment. Surgical resection is commonly used for early-stage oropharyngeal tumors, with postoperative radiotherapy and chemotherapy administered according to an individual’s clinical risk factors.^2^ However, >60% of patients with HNSCC present with locally advanced disease (stages III to IV) at initial diagnosis, and recurrence remains possible even after treatment (presentation, Li et al., 2024 Chinese Clinical Oncology Congress).

Cisplatin is currently the primary chemotherapeutic agent recommended in clinical guidelines for HNSCC.^3^ Its principal applications include radical standard treatment for locally advanced (stages III–IV) HNSCC, adjuvant concurrent chemoradiotherapy for patients with high-risk postoperative factors, such as positive surgical margins (R1) or lymph node extranodal extension (ENE); palliative chemotherapy for recurrent or metastatic (R/M) cases; and neoadjuvant treatment before radical surgery or radical chemoradiotherapy.^4^ Clinical research over the past five years supports the central role of cisplatin in concurrent chemoradiotherapy.^5^^,^^6^ In palliative and neoadjuvant therapy, immunotherapy shows broad application prospects, and combinations of cisplatin with immune checkpoint inhibitors have become an active area of clinical investigation^7^ (Table 1). Despite these advances, the overall five-year survival rate for HNSCC is only 50–60%,^8^ and the five-year survival rate for advanced and refractory diseases is >50%,^9^ necessitating effective treatment plans. The side effects and drug resistance also limit cisplatin application. Therefore, developing more effective, targeted therapies based on existing cisplatin treatment is warranted.

**Table 1:** Published clinical studies on cisplatin in the past five years

Primary content	Target population	Combination chemotherapy regimen	Main results	Reference
**Clinical Scenario 1: Definitive curative standard of care**				

TROG 12.01	126 patients with low-risk HPV-associated OSCC	cisplatin plus radiotherapy vs. cetuximab plus radiotherapy	cisplatin combined with radiotherapy remains the standard treatment for patients with low-risk HPV-associated oropharyngeal squamous cell carcinoma.	Hughes et al.^10^
KEYNOTE-412	804 patients with high-risk, unresectable LA-HNSCC	pembrolizumab plus cisplatin and radiotherapy vs. placebo plus cisplatin and radiotherapy	pembrolizumab combined with chemoradiotherapy does not significantly improve event-free survival.	Machiels et al.^6^
Avelumab-Related Study	treatment-naïve LA-HPSCC/OPSCC/LSCC	avelumab plus cisplatin and radiotherapy vs. cisplatin and radiotherapy vs. placebo plus cisplatin and radiotherapy	avelumab in combination with standard chemotherapy and radiotherapy fails to improve progression-free survival.	Lee et al.^11^
Comparative Study of Cetuximab vs. Cisplatin	407 patients with unresectable LA-HNSCC	cisplatin plus curative radiotherapy vs. cetuximab plus curative radiotherapy	concurrent cisplatin and radical radiotherapy remains the definitive standard of care for LA-HNSCC.	Hitt et al.^12^
ARTSCAN III	298 patients with stage III–IV LA-HNSCC	cetuximab plus chemotherapy vs. cisplatin plus chemotherapy	cetuximab is inferior to cisplatin in achieving local control when used in combination with RT for patients with LA-HNSCC.	Gebre-Medhin et al.^13^
Research on Maitake D for Alleviating the Toxicity of Chemotherapy and Radiotherapy	141 patients with advanced laryngeal and hypopharyngeal cancer	maitake D plus Cisplatin and Radiotherapy vs. Cisplatin and Radiotherapy	matsutake D-Concentrate can alleviate the adverse reactions associated with chemotherapy and radiotherapy, as well as improve quality of life.	Hu and Xu.^14^
Evaluation of the Combined Use of Nivolumab and Cisplatin	423 patients with newly diagnosed, non-metastatic, stage III/IV LA-HNSCC	nimotuzumab plus Cisplatin and Radical Radiotherapy vs. Cisplatin and Radical Radiotherapy	adding nimotuzumab to cisplatin-based chemoradiotherapy improves PFS, LRC, and DFS in patients with stage III/IV LA-HNSCC.	Menon et al.^15^
Phase I Study of Pacritinib Combined With Chemoradiotherapy	25 patients with LA-HNSCC	prexasertib plus Cisplatin and Radical Radiotherapy vs. prexasertib plus Cetuximab and Radical Radiotherapy	pacritinib can be safely combined with cetuximab and radiotherapy.	Yang et al.^16^
Evaluation of Therapeutic Efficacy: TPF Versus TPC Combined With Induction Chemotherapy	100 patients with locally advanced stage III or IV HNSCC	TPC vs. TPF+ICT	for patients with LA-HNSCC, TPC-containing ICT is an effective and well-tolerated one-day regimen.	Keil et al.^17^
TROG 12.01 (low-risk HPV)	522 patients aged 18 years and older with low-risk HPV-related OSCC	TOS and PORT vs. CRT	for patients with low-risk HPV-related OSCC, radiotherapy combined with cetuximab resulted in shorter failure-free survival without improving symptom burden or toxicity.	Rischin et al.^18^
Prophylactic Megestrol (MA) for Attenuating Chemoradiotherapy Toxicity	104 patients with advanced PLSCC receiving CCRT alone	MA initiated at the start of concurrent chemoradiotherapy vs. MA initiated after >5% weight loss	prophylactic MA significantly reduces weight loss and adverse events during chemoradiotherapy.	Lin et al.^19^
Phase II Efficacy Evaluation of Nimotuzumab Plus Cisplatin/Radiotherapy	37 patients with stage III/IV LA-HNSCC	cisplatin and Radiotherapy vs. iimotuzumab Cisplatin and Radiotherapy	nimotuzumab combined with cisplatin and radiotherapy is safe and highly effective in treating HNSCC	Ang et al.^20^

**Clinical Scenario 2: Postoperative adjuvant concurrent chemoradiotherapy**				

RTOG 9501/EORTC 22931	744 patients with HNC	CRT vs. RT	CRT significantly improves overall survival in patients with HNC.	Zumsteg et al.^21^
Randomized Phase II Trial of Docetaxel Versus Cisplatin	224 patients with postoperative high-risk LA-HNSCC	docetaxel plus Radiotherapy vs. cisplatin plus radiotherapy	docetaxel may be considered a concurrent chemoradiotherapy regimen for postoperative high-risk patients.	Jiang et al.^22^
Prospective Randomized Phase II Efficacy Evaluation of Docetaxel Plus Cisplatin Versus TPF	128 patients with LA-HNC	TL vs. TPF	for postoperative high-risk patients, the TL regimen may offer more significant clinical benefits with advantages in reducing toxicities, and can be considered a first-line treatment option.	Zhang et al.^23^

**Clinical Scenario 3: Palliative chemotherapy**				

Randomized Phase III Trial of Fintinib Plus Chemotherapy	870 patients with R/M-HNSCC	finotonlimab plus C5F vs. placebo plus C5F	finotrolimab plus C5F demonstrates significant overall survival advantage with acceptable safety in R/M HNSCC	Shi et al.^24^
Check Mate 651	947 patients with treatment-naive R/M- HNSCC	nivolumab plus ipilimumab vs. EXTREME	no superior survival benefit from dual immunotherapy over standard EXTREME chemotherapy, but it has better tolerability and more durable antitumor activity.	Hadded et al.^25^
KEYNOTE-669/ECHO-304	89 patients with treatment-naive R/M- HNSCC	pembrolizumab plus epacadostat vs. pembrolizumab vs. EXTREME	in R/M HNSCC, pembrolizumab plus epacadostat or monotherapy show comparable efficacy to EXTREME with less toxicity; early termination left the study underpowered to confirm the value of IDO1 inhibition.	Cho et al.^26^
KEYNOTE-048 (Japanese subgroup)	67 patients with treatment-naive R/M- HNSCC	pembrolizumab vs. pembrolizumab plus cisplatin and 5-fluorouracil	pembrolizumab monotherapy and pembrolizumab plus chemotherapy are effective and have manageable safety as first-line treatment for R/M HNSCC in Japanese patients	Takahashi et al.^27^
Efficacy of Pantoprazole Combined With Standard Chemotherapy	120 patients with advanced HNSCC	pantoprazole plus cisplatin vs. placebo plus cisplatin	adding pantoprazole to systemic therapy fails to improve survival outcomes or response rates in patients with advanced HNSCC; this combination is not recommended for clinical practice.	Noronha et al.^28^
CEFCID	180 patients with R/M- HNSCC	docetaxel plus CFP vs. CFP	in patients with R/M- HNSCC, treatment intensification with docetaxel does not adversely affect quality of life. However, patients receiving docetaxel experience more pronounced fatigue.	Goerling et al.^29^

**Clinical Scenario 4: Neoadjuvant therapy**				

Efficacy Evaluation of Camrelizumab Combined With TPF Induction Chemotherapy	51 patients with T3-4N0-3M0 stage LA HSCC	Camrelizumab plus TPF (All patients received the same regimen)	camrelizumab plus TPF induction chemoimmunotherapy demonstrate significant efficacy in LA HSCC.	Gong et al.^30^
Study of Pembrolizumab Plus Docetaxel and Cisplatin Therapy	52 patients with resectable LA-HNSCC	Pembrolizumab plus Docetaxel and Cisplatin (All patients received neoadjuvant immunotherapy)	the neoadjuvant regimen of pembrolizumab combined with docetaxel and cisplatin demonstrates high response rates, significant pathological responses, and favorable survival outcomes with manageable toxicity in resectable LA-HNSCC.	Wang et al.^31^
Efficacy Evaluation of Neoadjuvant Chemoimmunotherapy	48 patients with resectable LA-HNSCC	Camrelizumab plus Nab-paclitaxel and Cisplatin (All patients received neoadjuvant immunotherapy)	Camrelizumab combined with nab-paclitaxel and cisplatin demonstrate potential efficacy with acceptable safety in patients with resectable LA-HNSCC.	Wu et al.^32^
Neoadjuvant Cadonilimab Combined With Chemotherapy: A Phase II Study	30 patients with resectable LA-HNSCC	Cadonilimab plus Docetaxel and Cisplatin (All patients received neoadjuvant immunotherapy)	Cadonilimab plus neoadjuvant chemotherapy demonstrates favorable ORR and MPR with manageable toxicity in patients with HNSCC.	Cao et al.^33^
NeoRTPC02	28 treatment-naive patients with stage III-IVB HNSCC	low-dose radiotherapy plus tislelizumab, Nab-paclitaxel, and Cisplatin (All patients received neoadjuvant immunotherapy)	Neoadjuvant low-dose radiotherapy plus tislelizumab, nab-paclitaxel, and cisplatin achieve high pCR rates with manageable toxicity in locally advanced HNSCC, meeting the primary endpoint.	Liu et al.^34^
Randomized Phase II Study of Camrelizumab With or Without TPF	68 patients with stage III-IVA, LA-OSCC	camrelizumab vs. camrelizumab plus TPF	neoadjuvant camrelizumab combined with TPF chemotherapy demonstrates significantly superior pathological response rates and survival benefits compared with immunotherapy alone in LA-OSCC.	Liu et al.^34^
Phase II Study of Camrelizumab Plus Nab-Paclitaxel and Cisplatin	30 patients with resectable stage III-IVB HNSCC	Nab-paclitaxel (or docetaxel) and cisplatin plus camrelizumab (all patients received neoadjuvant immunotherapy)	neoadjuvant chemotherapy plus camrelizumab shows high ORR, pCR, and MPR with acceptable safety in locally advanced HNSCC.	Liu et al.^7^
OPHELIA	41 patients with operable HNSCC	cisplatin plus olaparib vs. olaparib vs. none vs. durvalumab plus olaparib	preoperative olaparib alone or combined with cisplatin or durvalumab is well tolerated and significantly modulates the tumor microenvironment in HNSCC.	Moutafi et al.^35^
TP vs. TPF (III期RCT)	495 adults with marginally resectable LA-OSCC	TP vs. TPF	in marginally resectable LA-OSCC TPF demonstrates significant survival advantages over TP, with increased but manageable toxicity.	Noronha et al.^36^
KCSG HN18-07	25 patients with LA-SNSCC	docetaxel plus Cisplatin and 5-Fluorouracil, with Pegfilgrastim (All patients received neoadjuvant immunotherapy)	neoadjuvant TPF chemotherapy with pegfilgrastim shows promising efficacy in unresectable locally advanced sinonasal squamous cell carcinoma, potentially facilitating organ function preservation.	Keam et al.^37^
NeoTGP01	23 patients with resectable LA-HNSCC	toripalimab plus GP (All patients received neoadjuvant immunotherapy)	toripalimab plus GP neoadjuvant immunochemotherapy is safe and feasible in resectable LA HNSCC.	Huang et al.^38^
Phase II Study of Toripalimab Plus Nab-Paclitaxel and Cisplatin	41 patients with resectable LA-OSCC	toripalimab plus Nab-Paclitaxel and Cisplatin vs. Nab-Paclitaxel and Cisplatin	neoadjuvant immunotherapy combined with chemotherapy improves pathological response rates and long-term survival outcomes in OSCC.	Wang et al.^39^

TROG, Trans-Tasman Radiation Oncology Group; OSCC, oral squamous cell carcinoma; LA-HNSCC, locally advanced head and neck squamous cell carcinoma; LA-HPSCC, locally advanced hypopharyngeal squamous cell carcinoma; LA-OPSCC, locally advanced oropharyngeal squamous cell carcinoma; LA-LSCC, locally advanced laryngeal squamous cell carcinoma; PFS, progression-free survival; LRC, locoregional control; DFS, disease-free survival; TPF, docetaxel (Taxotere), cisplatin, and 5-fluorouracil; TPC, docetaxel (Taxotere), cisplatin, and cyclophosphamide; PORT, postoperative radiation therapy; CRT, chemoradiotherapy; PLSCC, primary laryngeal squamous cell carcinoma; CCRT, concurrent chemoradiotherapy; OS, overall survival; TL, taxotere (docetaxel) plus cisplatin; TP, taxotere (docetaxel) plus platinum (cisplatin); LPC, laryngeal preservation chemotherapy; R/M-HNSCC, recurrent or metastatic head and neck squamous cell carcinoma; EXTREME, erbitux (cetuximab) in first-line treatment of REcurrent or MEtastatic head and neck cancer; CFP, cisplatin, 5-fluorouracil, and paclitaxel; LA HSCC, locally advanced hypopharyngeal squamous cell carcinoma; LA-SNSCC, locally advanced sinonasal squamous cell carcinoma; ORR, objective response rate; MPR, major pathological response; PCR, pathological complete response rate; GP, gemcitabine plus platinum (cisplatin).

PD-1 antibody-based neoadjuvant immunotherapy has prompted the reassessment of the role of non-tumor components of the tumor microenvironment (TME) in tumor progression, treatment resistance, and therapeutic efficacy (Table 1). CAFs were initially viewed primarily as physical barriers that prevent the penetration of therapeutic drugs, reducing treatment efficacy.^40^ Advances in research and the development of bioinformatics, proteomics, and metabolomics have provided novel insights into the role of CAFs in tumor progression and facilitated the development of treatments. For instance, CAFs secrete interleukin-6 (IL-6), which promotes STAT3 activation in tumor cells, enhancing proliferation.^41^ Surface expression of MHC I on CAFs affects the efficacy of tumor immunotherapy.^42^ CAFs, via paracrine mechanisms, enhance the colony-forming ability of HNSCC cells, potentially reducing the efficacy of cisplatin treatment.^43^ From a clinically oriented perspective, this review integrates emerging research trends to examine strategies for targeting CAFs with existing cisplatin-based treatment contexts. Such approaches can support the development of novel combination therapies and more effective treatments to improve patient prognosis and survival.

## The evolving role of CAFs: from matrix support to resistance to cisplatin treatment

### Understanding of the characteristics and functions of CAFs has deepened over time

In 1994, Welt et al. introduced the term *carcinoma-associated fibroblasts* to describe “fibroblasts from cancer lesions”, although the concept lacked functional or mechanistic definitions.^44^ That same year, another study reported functional differences between fibroblasts derived from normal breast tissue and those isolated from breast cancer tissue.^45^ Nevertheless, for many years, tumor-associated fibroblasts were largely regarded as stromal cells and were not clearly distinguished from normal fibroblasts. Although some studies suggested that “tumor-derived fibroblasts” possess tumor-promoting properties,^46^ they are generally still considered stromal cells.

In 2006, the article *Fibroblasts in Cancer* explicitly used the term “cancer-associated fibroblasts (CAFs)” and defined them as an important subtype of fibroblasts.^47^ During the same period, other studies showed that fibroblasts could promote tumor formation^48^ and prompted exploration of CAF-targeted strategies for antitumor therapy.^49^ Subsequent research demonstrated that fibroblasts in the TME are heterogeneous, and using αSMA or desmin alone as markers cannot identify all CAFs.^50^ The diversity of CAF sources, including LFs,^51^ endothelial cells,^52^ myeloid cells,^53^ and adipose-derived stem cells,^54^ supports this heterogeneity. Preclinical and clinical studies subsequently showed that CAFs contribute to chemotherapy resistance, reduce tumor cell sensitivity to paclitaxel,^55^^,^^56^ promote resistance to sorafenib treatment in prostate cancer,^57^ and define a CAF subset in breast cancer with extremely low activated ERK signaling, associated with a poor response to tamoxifen treatment and adverse cancer prognosis.^58^^,^^59^^,^^60^^,^^61^

Podoplanin expression in CAFs has been proposed as an unfavorable prognostic marker in invasive ductal carcinoma.^62^ In addition, several drugs and small molecules have been identified that target CAFs, such as dasatinib, which partially reverses the cancer-associated fibroblast phenotype, restoring it to a normal fibroblast-like phenotype and alleviating disease progression.^63^ By approximately 2010, CAF research had remained relatively limited, but their heterogeneity, tumor-promoting function, contribution to poor chemotherapy response, and therapeutic potential had become increasingly evident. Targeting CAFs for treatment is a promising clinical strategy.

After 2011, CAF research shifted from the periphery to the forefront of tumor biology. CAFs promote angiogenesis,^64^^,^^65^^,^^66^ enhance ECM stiffness,^67^ influence tumor-immune cell functions,^68^^,^^69^^,^^70^ and affect responsiveness to chemotherapy drugs,.^71^^,^^72^^,^^73^ Mechanistic studies further clarified how CAFs communicate with tumor cells and other components of the microenvironment through metabolic reprogramming,^74^ paracrine signaling,^75^ and other mechanisms. In clinical translation, researchers began systematically evaluating the prognostic value of CAF markers. For example, Cav-1 expression levels predict survival in patients with breast cancer and gastric cancer.^76^^,^^77^ Therapeutic strategies targeting CAFs have also become more diverse, with FAP-activated prodrugs reaching the proof-of-concept stage (Figure 1).^78^

**Figure 1:**
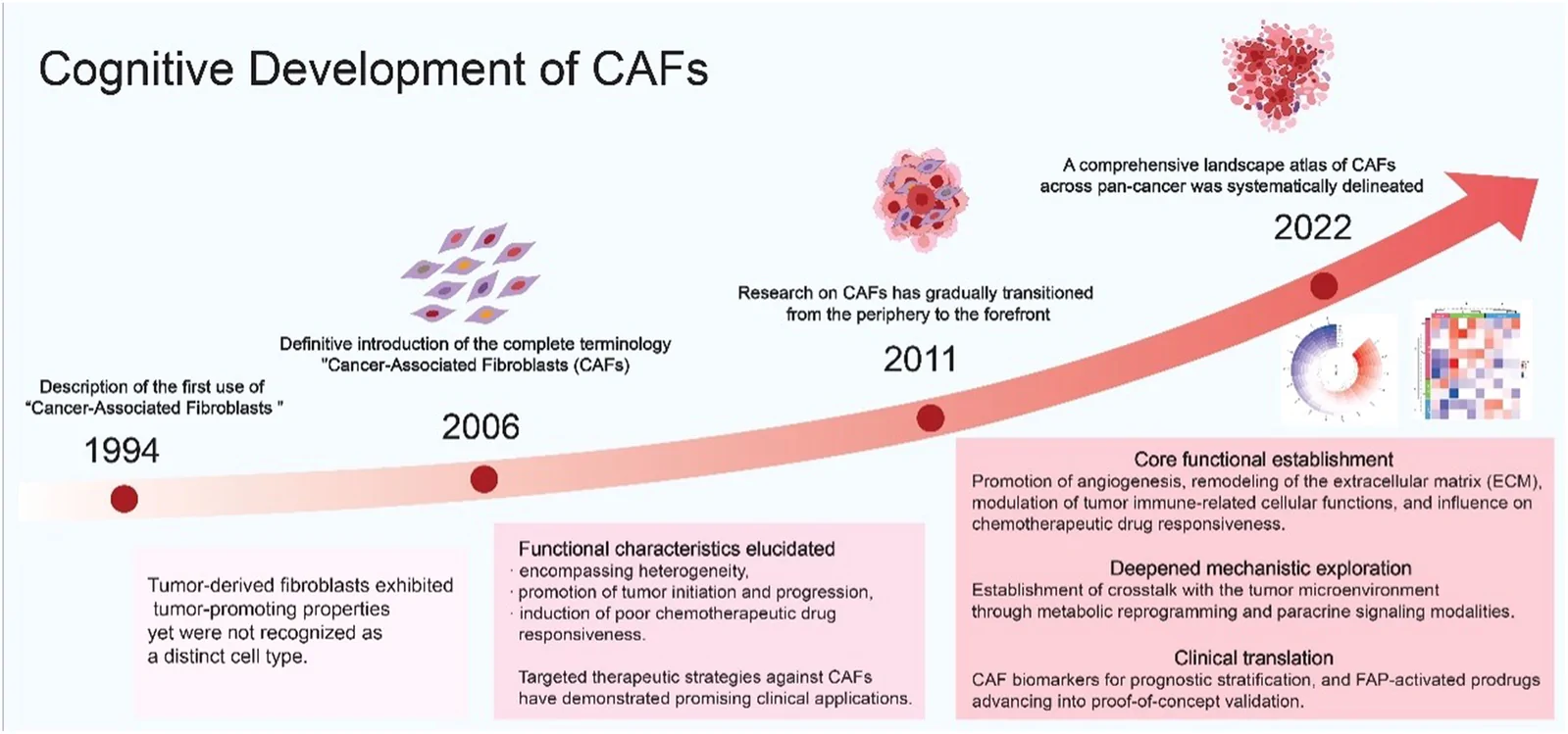
The understanding of CAF characteristics and functions has deepened over time

CAF heterogeneity complicates research. CAFs differ across tumor types and even among samples from the same cancer type.^79^ Defining CAFs and characterizing the functions of related cell subpopulations has always been challenging. Bioinformatics has helped address this issue by enabling the identification of CAF subsets with distinct markers and functional properties. For example, Costa et al. identified CAF subpopulations expressing markers such as CD10 and GPR77 and defined them as having immune regulatory functions.^80^ Han et al. discovered antigen-presenting CAFs (apCAFs), a novel CAF subpopulation in pancreatic ductal adenocarcinoma that expresses MHC class II molecules and activates CD4^+^ T cells.^81^ In 2022, Luo et al. systematically mapped the landscape of CAFs across 16 cancer types via pan-cancer single-cell sequencing and categorized CAFs into iCAF, myCAF, and matCAF. The authors also explained their functional characteristics and markers. Myofibroblast-like CAFs (MyCAFs), which highly express contraction-related genes, such as α-SMA and TAGLN, dominate ECM remodeling and fibrosis and are located at the tumor invasion front. Inflammatory CAFs (iCAFs) secrete inflammatory factors, such as IL-6 and CXCL12, and promote tumor inflammation and angiogenesis. iCAFs are also distributed in the central region of the tumor. Antigen-presenting CAFs (apCAFs) express MHC class II molecules (e.g., HLA-DRA and CD74), possess antigen-presenting capabilities, and regulate T cell immune responses.^82^ This work overcame limitations imposed by individual cancer types and sample-specific CAF heterogeneity, establishing a universal conceptual framework and providing an in-depth explanation of CAF plasticity. The work also reveals the enrichment and prognostic relevance of specific CAF subtypes in certain cancer types, providing molecular evidence for precision therapy strategies targeting CAF subtypes across different cancers. The findings and methods in this review will inform subsequent research.

### Similarities and differences in CAF subtypes associated with cisplatin resistance in HNSCC and pan-cancer

Pan-cancer research has provided valuable insights into CAFs in HNSCC and identified iCAF, myCAF, and matCAF in numerous HNSCC studies.^83^ Furthermore, single-cell sequencing technology has revealed specific functional CAF subpopulations in HNSCC. CAFs’ impact on chemotherapy response has been well documented in HNSCC.^84^^,^^85^^,^^86^^,^^87^ For instance, studies have identified a CAF-promoting subtype in OSCC, characterized by spatial heterogeneity among CAF subpopulations, that can reduce sensitivity to cisplatin treatment.^84^

#### CAF-P Enhances Cisplatin Resistance in OSCC Through Paracrine Signaling

Promoting CAFs (CAF-P)-derived exosomes specifically enrich microRNA-876-3p, which targets and inhibits the transcription factor GATA1, downregulating the expression of insulin-like growth factor-binding protein 3 (IGFBP3) (Figure 2). IGFBP3 inhibits cell proliferation and promotes apoptosis; its absence significantly reduces the sensitivity of OSCC cells to cisplatin, and its expression is mechanistically promoted by GATA1, a direct target of miR-876-3p. Treatment with CAF-P exosomes carrying anti-miR-876-3p reduced cisplatin resistance.^84^

**Figure 2:**
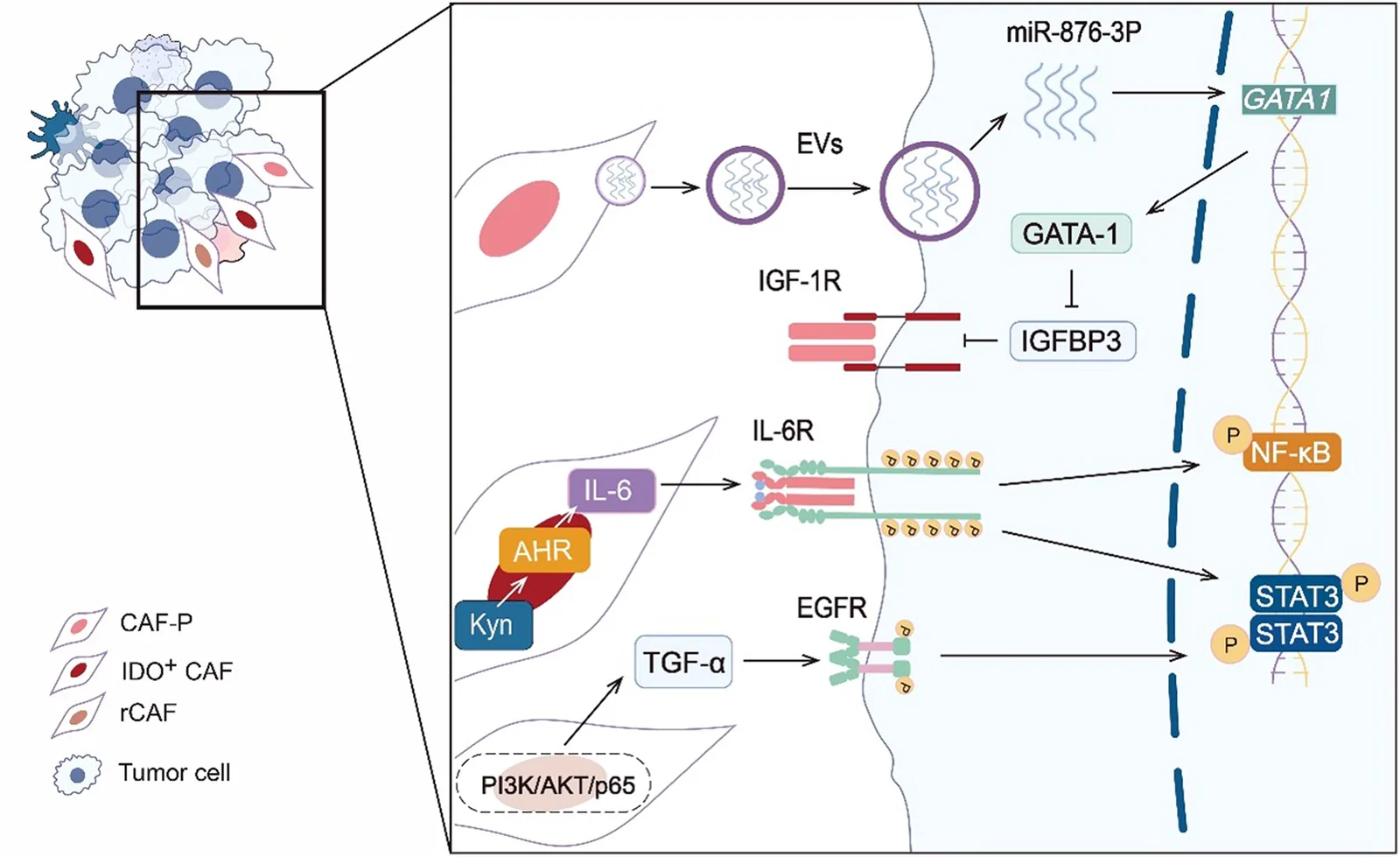
CAF subtypes associated with cisplatin resistance in HNSCC tumor cells ① CAF-P-derived extracellular vesicles suppress GATA1 expression in tumor cells, thereby reducing expression of the downstream target IGFBP3, which is positively associated with cisplatin sensitivity. ② IDO1^+^ CAFs release kynurenine, activate the aryl hydrocarbon receptor, enhance IL-6 secretion, and stimulate the p-STAT3/NF-κB signaling pathway, contributing to cisplatin resistance. ③ In chemotherapy-resistant CAFs, activation of the PI3K/AKT/p65 pathway promotes cell survival and cytokine production, further enhancing cisplatin resistance.

#### IDO1^+^ CAFs promote tumor cell invasiveness while enhancing cisplatin resistance in HNSCC

IDO1-expressing CAFs synthesize kynurenine, the tryptophan metabolite that activates the aryl hydrocarbon receptor and induces IL-6 release, a pro-inflammatory cytokine (Figure 2). IL-6 enhances the p-STAT3/NF-κB signaling pathway, thereby promoting tumor cell proliferation and conferring resistance to cisplatin. HNSCC cells co-cultured with IDO1^+^ CAFs exhibit enhanced invasive capacity and greater cisplatin tolerance, both *in vitro* and *in vivo*.^86^^,^^87^

#### Chemotherapy-resistant CAFs (rCAFs) activate the PI3K/AKT/p65 signaling pathway to enhance cisplatin resistance

rCAFs arise from a subtype of CAFs isolated from cisplatin-resistant HNSCC clinical samples with high tolerance to chemotherapy-induced apoptosis associated with the activation of the PI3K/AKT/p65 signaling pathway, thereby promoting cell survival and cytokine synthesis. One such cytokine is TGF-α, which is released by rCAFs and activates the EGFR/Src/STAT3 survival signaling pathway (Figure 2). This pathway enhances cancer cells' tolerance to chemotherapy.^85^

Given CAFs’ highly heterogeneous nature, we compared the CAF subpopulations associated with cisplatin resistance in HNSCC with those in other cancer types to characterize them (Table 2). Multiple CAF subtypes show activation of tumor cell proliferation-related pathways, including STAT3 (IL-6/JAK-STAT3 and EGFR/Src/STAT3), PI3K/AKT/mTOR, and MAPK (Figure 2).^85^^,^^88^^,^^89^ In HNSCC and other cancer types, CAFs influence tumor cell responsiveness to cisplatin via paracrine signaling.^84^^,^^90^^,^^91^ Specifically, in liver cancer, cisplatin stimulates tumor cells to promote CAF formation through paracrine signaling, thereby inducing treatment resistance.^92^ Some cisplatin-resistant CAF subpopulations exhibit characteristics of metabolic reprogramming. For instance, IDO1^+^ CAFs in HNSCC synthesize and secrete kynurenine, activating the aryl hydrocarbon receptor.^86^^,^^87^ In gastric cancer, PPIC-labeled CAFs have been linked to glucose metabolism.^93^ In HNSCC and colon cancer, rCAFs are distinct subtypes. This highlights the limitations in defining CAFs solely by function.^85^^,^^94^ Meanwhile, some studies have only observed an association between a specific subset of CAFs and resistance to cisplatin treatment, without further exploration.

**Table 2:** Comparison of CAF subtypes associated with cisplatin resistance in HNSCC and pan-cancer

Cancer type	Classification	Mechanism and pathway	Reference
**CAF subtypes associated with cisplatin resistance in HNSCC**			

HNSCC	promoting CAFs (CAF-P)	the paracrine release of microRNA 876-3p-rich vesicles suppresses the expression of genes that sensitize tumor cells to cisplatin, such as GATA1 and IGFBP3, leading to enhanced resistance to cisplatin.	Kang et al.^84^
HNSCC	IDO1^+^ CAFs	synthetic kynurenine pathway can activate the aryl hydrocarbon receptor, thereby releasing interleukin-6 (IL-6) and promoting tumor cell proliferation, as well as conferring resistance to cisplatin.	Chen et al and Choi et al.^86^^,^^87^
HNSCC	chemotherapy-resistant CAFs (rCAFs)	cancer-associated fibroblast (CAF) subtypes derived from cisplatin-resistant cells release TGF-α, which activates the EGFR/Src/STAT3 survival signaling pathway and enhances tolerance to chemotherapy in cancer cells.	Su et al.^85^

**CAF subtypes associated with cisplatin resistance in HNSCC in pan-cancer**			

Liver cancer	COL1A1^+^ and ACTA2^+^ CAFs	cisplatin induces tumor cells to produce excessive ROS, which promote the transformation of hepatic stellate cells into CAFs via a paracrine mechanism. CAFs reduce the efficacy of chemotherapy drugs. The PI3K signaling pathway is a key driver of HSC/CAF activation.	Ren et al.^92^
Non-small-cell lung cancer (NSCLC)	patient-derived BM-CAFs	promotes epithelial-mesenchymal transition (EMT) and cancer stem cell (CSC) phenotypes. CAFs secrete IL-26 and CX3CL1 signals. IL-26 activates the JAK-STAT3 pathway, while CX3CL1 activates the JAK-STAT3 and AKT-mTOR pathways. It drives tumor progression and treatment resistance.	Salam et al.^88^
Osteosarcoma	Rab27a^+^ CAFs	suppress CREBRF and activate the MAPK pathway, thus promoting tumor progression and chemotherapy resistance.	Salam et al.^89^
Gastric cancer	PPIC-labeled CAFs	associated with cisplatin resistance and poor prognosis, and may contribute to drug resistance by affecting signaling pathways, such as glucose metabolism and ECM remodeling. The precise mechanisms require further investigation.	Yin et al.^93^
Breast stromal	E2F1-expressing fibroblasts	fibroblasts that express high levels of E2F1 make breast cancer cells more resistant to chemotherapy drugs. Fibroblasts that lack E2F1 make breast cancer cells more sensitive to cisplatin.	Al-Kharashi et al.^95^
Non-small-cell lung cancer (NSCLC)	CD248^+^ CAFs	activation of the CD248–NF-κB axis by CAFs promotes the secretion of IL-8, thereby inducing chemotherapy resistance in NSCLC.	Wu et al.^91^
Gastric cancer	GFPT2 high expression CAFs	expression of *GFPT2* mRNA correlates with sensitivity to multiple chemotherapeutic agents, including docetaxel, paclitaxel, and cisplatin. It may serve as a biomarker for assessing prognosis and immune infiltration in gastric cancer.	Yang et al.^96^
Colorectal cancer	chemotherapy-resistant CAF s (rCAF)	exosomes secreted by R-CAFs promote the progression of colorectal cancer and resistance to cisplatin by transmitting VEGFA.	Shi et al.^94^
Pancreatic cancer	STAR^+^ CAF	CAFs with high SCD expression significantly enhance pancreatic cancer cell resistance to cisplatin and gemcitabine.	Wu et al.^97^

IDO1, indoleamine 2,3-dioxygenase 1; ROS, reactive oxygen species; SPP1, secreted phosphoprotein 1.

Consensus on the conceptual definition of CAF subtypes associated with cisplatin resistance in HNSCC has yet to be reached. First, unified definitions and classification criteria for CAF subtypes are lacking: Some are identified by function, others are derived from bioinformatics analyses, and some stem from anatomical heterogeneity. Second, most studies have reported only associations between specific CAF subgroups and cisplatin resistance, with no clear evidence of a causal relationship. Furthermore, existing studies predominantly consist of static observations that do not track the dynamic evolution of CAF subpopulations during cisplatin therapy. To address these gaps, future studies should integrate single-cell transcriptomics, spatial transcriptomics, and ATAC-seq data from HNSCC, among other cancer types, to establish a consensus classification and a core set of signature genes for cisplatin-resistant CAFs across cancer types. Longitudinal clinical cohorts should also be designed to collect tumor samples before, during, and after treatment, as well as at the time of recurrence. Combining single-cell multi-omics and pseudo-time-series analysis could reconstruct the translational trajectories of CAF subpopulations.

## CAFs modulate cisplatin response via signaling pathways and physical barriers in HNSCC

CAFs promote tumorigenesis and progression by reprogramming metabolism, signaling paracrine factors, remodeling the ECM, promoting angiogenesis, and suppressing the immune system. They also establish a complex, functional symbiotic relationship with HNSCC cells that significantly affects tumor cell sensitivity to cisplatin.^84^^,^^86^^,^^87^^,^^98^^,^^99^^,^^100^^,^^101^^,^^102^^,^^103^^,^^104^ This review systematically elucidates the mechanisms underlying CAF-mediated cisplatin resistance in HNSCC and the research progress in this area.

### Cisplatin treatment sensitivity is influenced by paracrine signaling from HNSCC-associated CAFs and tumor cells

Paracrine signaling, the primary mechanism by which CAFs promote cisplatin resistance in HNSCC, involves soluble cytokines, growth factors, and functional nucleic acids delivered via exosomes. These signaling molecules activate survival pathways in cancer cells, inhibit cisplatin-induced apoptosis, promote metabolic reprogramming, and minimize cisplatin’s cytotoxic effects.

#### CAFs secrete soluble factors that increase the resistance of tumor cells to cisplatin

CAFs primarily mediate cisplatin resistance by activating autophagy. In tongue squamous cell carcinoma (TSCC), *in vivo* and *in vitro* studies have demonstrated that CAFs secrete soluble factors that significantly increase the expression of LC3-II and Beclin1, autophagy markers, in TSCC cells, thereby activating autophagic flux and protecting cancer cells from cisplatin-induced apoptosis.^105^ Another study found that stromal α-SMA expression increased in HNSCC samples following chemotherapy. High expression of the autophagy protein p62 in cancer cells is also closely associated with the activation of stromal CAFs following chemotherapy.^106^ Stromal α-SMA expression increases in HNSCC samples following chemotherapy, and high p62 expression in cancer cells is closely associated with the activation of stromal CAFs (Figure 3). Additionally, CAFs in HNSCC exhibit higher basal autophagy levels than normal fibroblasts.^107^

**Figure 3:**
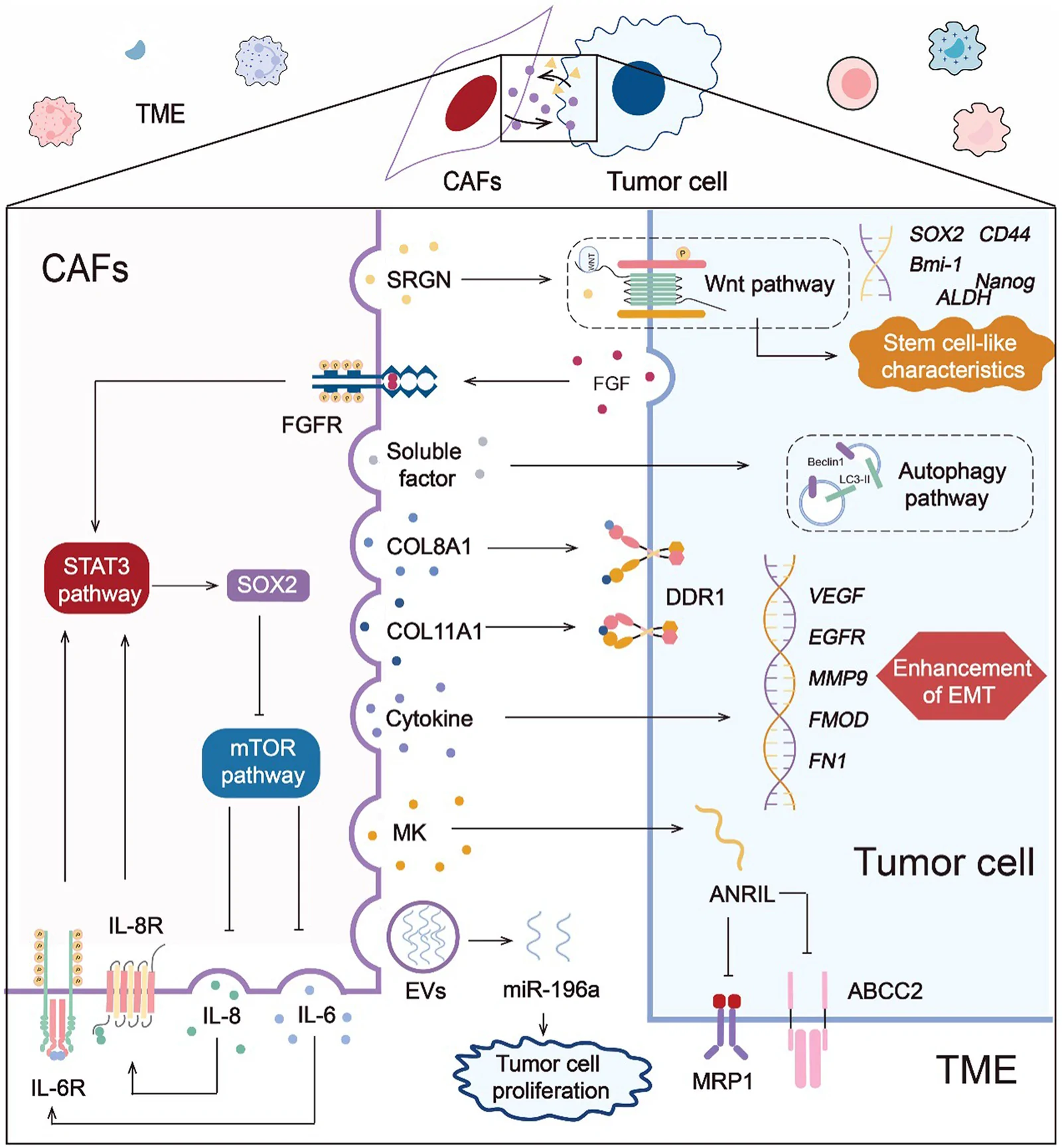
Interactions between CAFs and tumor cells modulate cisplatin resistance in HNSCC ① CAF-derived FGF5 activates FGFR2/NRF2 signaling, inhibits ferroptosis, and reduces NPC sensitivity to cisplatin. ② CAF-secreted midkine (MK) increases ANRIL expression in tumor cells, impairing MRP1 and ABCC2 drug transporter function. ③ CAFs from some samples induce cisplatin-resistant phenotypes in HNSCC tumor cells, characterized by increased VEGFA, PGE2S, COX2, EGFR, and NANOG expression. CAFs from other samples increase HNSCC tumor cell sensitivity to cisplatin, as indicated by increased CCL2 expression. ④ CAF-derived exosomes transfer functional miRNA-196a to HNC cells, promoting chemoresistance and invasive behavior. ⑤ Tumor cell-derived FGF activates FGFR/STAT3/SOX2 signaling in CAFs, suppresses mTOR signaling, restores IL-6 and IL-8 secretion, and maintains the tumor-promoting CAF phenotype. ⑥ COL8A1 and COL11A1 activate DDR1, reducing tumor-cell apoptotic responses to cisplatin.

In nasopharyngeal carcinoma, CAFs secrete large amounts of fibroblast growth factor 5 (FGF5), which binds to fibroblast growth factor receptor 2 (FGFR2) on cancer cell surfaces, thereby activating the KEAP1/NRF2/HO-1 pathway, inhibiting cisplatin-induced ferroptosis, and reducing chemosensitivity (Figure 3). Targeting the FGF5/FGFR2 axis has been shown to enhance cisplatin efficacy.^90^ Primary CAFs isolated from patients with OSCC secrete high levels of Midkine (MK), which eliminates cisplatin-induced tumor cell death. MK promotes cisplatin resistance by increasing ANRIL expression in tumor cells, thereby inhibiting the function of the drug transporters MRP1 and ABCC2; this effect is dependent on caspase-3/BCL-2.^108^

#### *C*AFs secrete cytokines that affect tumor cell metabolic reprogramming and cisplatin resistance in HNSCC

CAF-secreted cytokines affect tumor cell metabolic reprogramming. For example, HGF, a key factor mediating cisplatin resistance, activates the PI3K/AKT and MAPK/ERK pathways by binding to the c-Met receptor on cancer cell surfaces, thereby inhibiting cisplatin-induced apoptosis (Figure 3). Furthermore, by increasing lactate production, HGF has been shown to induce metabolic reprogramming in HNSCC cells, forming a “metabolic symbiosis” pattern.^109^ In an OSCC study, C-X-C motif ligand 1 (CXCL1) from CAFs downregulated DEC2 expression in OSCC cells, ultimately awakening dormant cells and leading to tumor recurrence. DEC2 overexpression enhances the invasive and migratory abilities of OSCC cells, while inhibiting their proliferation and glucose metabolism. This drives cells into a dormant, cisplatin-resistant state.^110^

#### CAFs influence drug-resistance-related protein expression in HNSCC cells through paracrine signaling

Co-culturing HNSCC cells with CAFs increases the expression of vascular endothelial growth factor A (VEGFA), prostaglandin E2 synthase (PGE2S), cyclooxygenase 2 (COX2), epidermal growth factor receptor (EGFR), and NANOG, which are associated with increased resistance characteristics (Figure 3). Meanwhile, C-C motif chemokine ligand 2 (CCL2) expression is linked to sensitization effects.^43^ In another study, CAFs significantly enhanced EGFR expression, which ultimately affected epithelial-mesenchymal transition (EMT) and cancer stem cell (CSC) phenotypes and influenced the efficacy of cisplatin treatment.^99^

#### CAF-derived exosomes mediate cisplatin resistance in HNSCC cells

Exosomes are key carriers via which CAFs transmit drug-resistance signals to HNSCC cells. Exosomal microRNAs (miRNAs) can stably regulate gene expression in tumor cells and promote the development of drug resistance. Chemicals derived from CAFs can transfer functional miR-196a to head and neck cancer cells, conferring chemotherapy resistance and an invasive phenotype. hnRNPA1 facilitates the encapsulation of miR-196a within extracellular vesicles (EVs) secreted by CAFs. In the HNC microenvironment, miR-196a interacts with CDKN1B and ING5, promoting cell proliferation while suppressing apoptosis (Figure 3). Depleting CAF-derived exosomes or extracting miR-196a from them restored cisplatin sensitivity in HNC cells. Clinically, elevated plasma miR-196a levels correlate with reduced overall survival and diminished drug sensitivity, suggesting that miR-196a may serve as both a predictor of cisplatin resistance in head and neck cancer and a potential therapeutic target.^104^

#### Tumor cells induce cisplatin-resistant phenotypes in CAFs through paracrine signaling

The binding of FGF, a factor released by tumor cells, to FGFR on CAFs initiates SOX2 transcription via the STAT3 pathway, thereby inhibiting mTOR and its downstream effector proteins. This promotes the release of IL-6 and IL-4, which bind to FGFR, initiating SOX2 transcription in CAFs via the STAT3 pathway (Figure 3). These cytokines are produced through an autocrine positive feedback mechanism, reinforcing the CAF phenotype.^107^

### CAFs in HNSCC remodel the ECM to impede cisplatin penetration

The ECM serves as a scaffold for cells and molecules and a dynamic signaling platform. CAFs influence cisplatin delivery and cancer cell sensitivity to chemotherapy by secreting collagen, modifying ECM components, and forming physical barriers.^111^

#### CAF-mediated collagen deposition and matrix stiffening impede cisplatin penetration

CAFs secrete various collagens, including COL1A2, COL3A1, and COL5A2, which are significantly upregulated in HNSCC. The COL8A1 and COL11A1 proteins activate the DDR1 receptor (Figure 3), the absence of which can inhibit collagen’s pro-growth, pro-migration, and pro-drug-resistance effects on HNSCC cells.^101^ Increased collagen deposition increases tumor stroma hardness and promotes dense connective tissue hyperplasia, hindering the penetration of small-molecule drugs, such as cisplatin, into deeper tumor regions, thereby reducing the effective drug concentration.^100^

#### CAF-derived cytokines associated with ECM remodeling and cisplatin resistance

The cytokines produced by CAFs significantly enhance the expression of specific genes in HNSCC cells (Figure 3). Among them, the expression of the matrix metalloproteinase 9 (MMP9) and fibromodulin (FMOD) genes is associated with drug resistance in HNSCC cells.^98^ MMP9 and FMOD are important ECM components in HNSCC. MMP9 plays a potentially key role in OSCC cell metastasis and invasion,^112^ and FMOD is overexpressed in OSCC and significantly associated with malignant progression and lymph node metastasis.^113^

In other cancer types, ECM physical properties can regulate chemotherapy sensitivity through mechanosensitive signaling. For example, in breast cancer lung metastasis, a soft ECM induces mitochondrial fission via mechanosensitive signaling, thereby activating the mtROS-NRF2 antioxidant axis and enhancing tumor cell resistance to ROS-dependent chemotherapeutic agents, such as cisplatin. Inhibiting DRP1 or NRF2 reverses soft-matrix-mediated cisplatin resistance.^114^ Dang et al. have reported that the FAK/YAP mechanosensory axis promotes cisplatin resistance in ovarian cancer. Resistant cancer cells exhibit high FAK expression and a “mechanically stiff” phenotype. Excessive FAK activation promotes YAP nuclear translocation by increasing nuclear membrane tension, thereby inhibiting apoptosis. In animal models, combining defactinib, an FAK inhibitor, with cisplatin significantly inhibited tumor growth.^115^ These findings inform the development of treatment strategies for cisplatin resistance in HNSCC.

Existing evidence suggests that the mechanisms by which CAFs promote cisplatin resistance in HNSCC remain to be fully elucidated.^43^^,^^98^^,^^99^ Functional studies have shown that CAFs affect the resistance of HNSCC cells to cisplatin. However, most current studies focus primarily on interactions between CAFs and tumor cells,^84^^,^^86^^,^^87^^,^^98^^,^^99^^,^^100^^,^^101^^,^^102^^,^^103^^,^^104^ and only a few have addressed the impact of CAFs on ECM mechanical remodeling and cisplatin resistance.^98^^,^^100^^,^^101^ Research on the interactions between CAFs and other components of the TME that affect cisplatin treatment in HNSCC is lacking. However, CAFs interact with other components of the TME through dynamic regulatory networks in esophageal squamous cell carcinoma,^116^ ovarian cancer,^117^ and colorectal cancer.^118^ These networks encompass biochemical signal transduction, mechanical force effects, metabolic reprogramming, and immune regulation. In HNSCC, most studies focus on tumor cell proliferation to demonstrate that CAFs influence the response to cisplatin treatment by affecting proliferation, invasion, migration, and related signaling pathways.^43^^,^^84^^,^^86^^,^^87^^,^^98^^,^^99^^,^^100^^,^^101^^,^^102^^,^^103^^,^^104^^,^^119^ In contrast, research on factors such as HPV infection, ECM remodeling, mechanical forces, and immune cell infiltration is scarce.

The mechanisms by which CAFs promote cisplatin resistance in HNSCC remain unclear; therefore, future research should incorporate additional TME components rather than focusing solely on interactions between CAFs and tumor cells. Second, using techniques such as three-dimensional (3D) scaffolds with controllable stiffness and drug-penetration tracing, future studies should determine how CAFs mechanically remodel the ECM and assess their impact on the physical barrier to cisplatin. Additionally, the research focus should shift from the effects of CAFs on tumor proliferation to the core mechanisms of resistance, which will require identifying key molecular pathways underlying resistance rather than merely exploring correlations.

## Development and advances in research models of cisplatin -resistant HNSCC-associated CAFs

The spatiotemporal heterogeneity of CAFs presents substantial challenges for research. Key issues include accurately labeling CAFs with specific functions in tumor samples, identifying factors associated with CAF functions, and establishing *in vitro* and *in vivo* experimental models that maintain CAF specificity (phenotype) to enable more in-depth research. Advances in bioinformatics and experimental technologies have addressed some of these challenges, advancing basic CAF research and opening new possibilities.

### Multi-omics and bioinformatics approaches advance understanding of CAFs in HNSCC

Current research uses bioinformatics, genomics, proteomics, or combinations to analyze CAF characteristics and identify CAF subtypes with specialized functions in tumor samples from diverse sources. The characterization of CAFs has shifted from single-transcriptomic descriptions to an integrated, multi-omics approach to elucidate the regulatory mechanisms and functional states underlying CAF heterogeneity. This research paradigm primarily uses single-cell multi-omics to analyze the transcriptional dynamics and epigenetic regulation of CAF subpopulations, and spatial multi-omics to characterize the spatial distribution patterns and cellular interaction networks of CAFs within the TME. This approach builds a comprehensive chain of evidence spanning from “molecular characteristics” to “spatial functions”.

#### Genomic screening identifies high COL5A2 expression in CAFs from erlotinib-resistant HNSCC

Gene expression profiling analyses of HNSCC cell lines that were both sensitive and resistant to erlotinib using the GEO and TCGA databases identified 124 differentially expressed genes. COL5A2 was significantly upregulated in resistant samples and highly expressed in CAFs, and the erlotinib resistance score positively correlated with the degree of CAF infiltration.^120^

#### Multiomics-defined CAF subsets associated with cisplatin sensitization

Comparing single-cell transcriptomic data from chemotherapy-sensitive and -resistant patients can identify gene sets significantly associated with the resistance phenotype. Spatial transcriptomics can precisely map the spatial distribution of specific CAF subpopulations, while cell-to-cell communication analysis can be employed to infer their functional interaction networks. Validation is conducted through independent cohorts and functional experiments.

Significant progress has been made in identifying cisplatin-resistance-associated CAF subtypes across multiple cancer types (Table 2). In serous ovarian cancer, researchers have integrated scRNA-seq data from chemotherapy-sensitive and -resistant patients and identified the STAR^+^ CAF subpopulation as significantly enriched in the sensitive group. Spatial transcriptomics and multiplex immunofluorescence confirmed that this subpopulation is distributed around tumor cells, and ligand-receptor analysis suggested that it may enhance chemotherapy sensitivity by inhibiting WNT signaling.^121^

Identifying CAF subtypes associated with cisplatin resistance continues to face several challenges. Heterogeneity among patients can obscure genuine differences between chemotherapy-sensitive and resistant groups, necessitating validation in larger cohorts. The cell-to-cell interactions suggested by spatio-omics are currently only predictive and require causal validation through *in vitro* co-culture or *in vivo* gene editing models. Most studies collect samples only at a single time point, limiting their ability to track the dynamic evolution of CAF subtypes under chemotherapy stress. Future research should integrate longitudinal sampling, including single-cell and spatio-genomic data collected before, during, and after treatment, as well as at the time of recurrence, to more comprehensively map the evolutionary trajectories of CAF subtypes associated with resistance (Figure 4).

**Figure 4:**
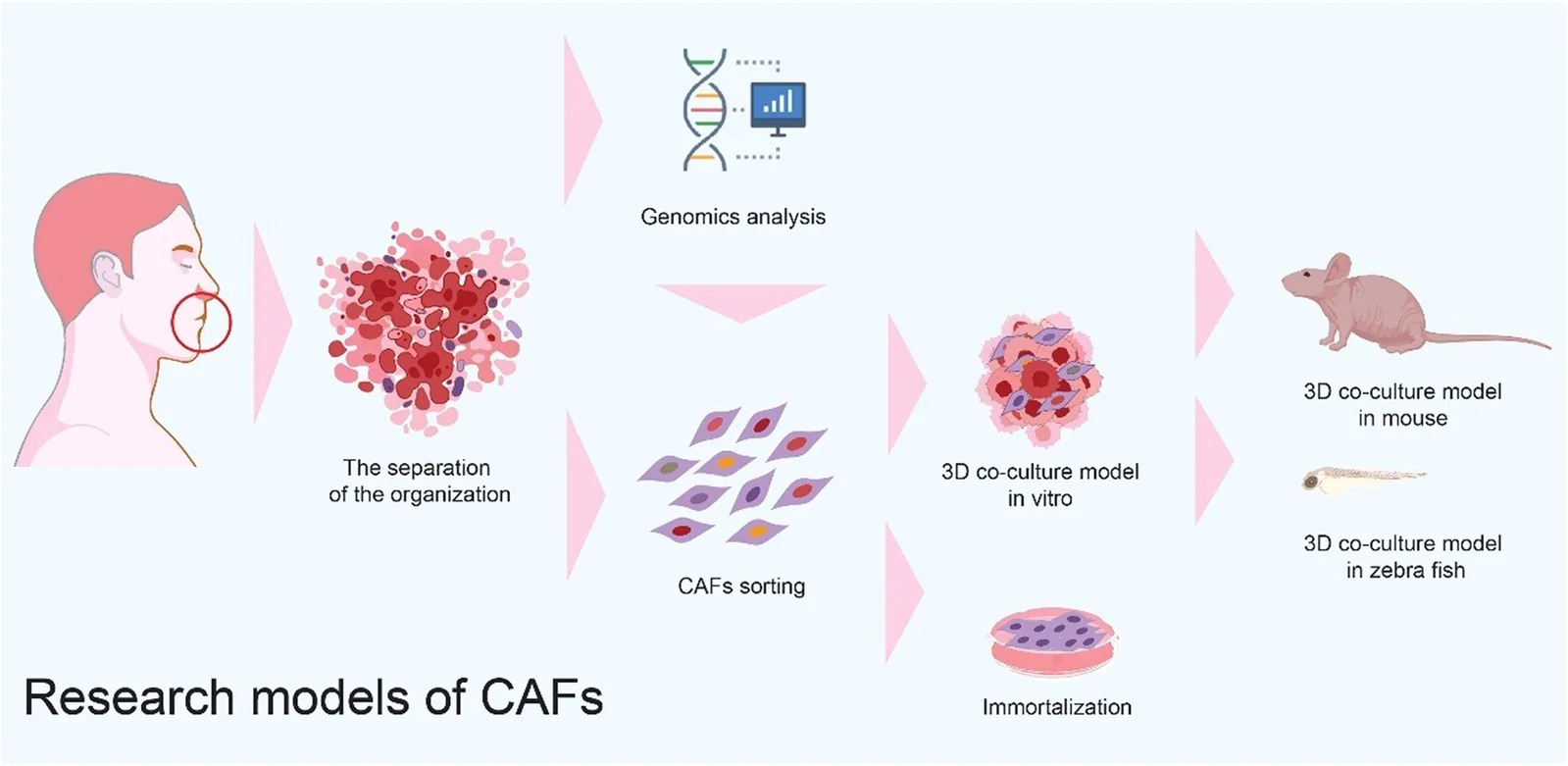
Research models for studying cisplatin resistance

### Development of experimental models for CAF-associated cisplatin resistance

The evolving TME affects the heterogeneity and phenotypic plasticity of CAFs. To determine the function of CAFs, complex organoid models that integrate patient-derived CAFs, immune cells, and tumor cells should be developed along with engineered ECM in conjunction with genetically engineered mouse models (GEMMs) to systematically and accurately examine the mechanisms of CAF state transition and validate its efficacy.^122^ Currently, the correlation between cisplatin resistance and CAFs in HNSCC is being investigated. Scholars have developed a “3D sheet” *in vitro* model of drug sensitivity to more precisely mimic the TME and tissue architecture^123^^,^^124^ and have utilized a spheroid model of tumor cells co-cultured with primary CAFs for animal modeling to enhance drug screening (Figure 4).^123^^,^^125^

#### Isolation of CAFs and establishment of immortalized cell lines

Successfully isolating and purifying high-purity CAFs from tissues is an important step in research. Huang et al. have developed a gentle and efficient method of separation. First, the authors thoroughly chopped fresh tumor tissue, digested it with trypsin, treated it with type IV collagenase three times, and purified the samples using FAP magnetic beads. The isolated CAFs are cultured in a medium, and their surface expression of FAP is detected (Figure 4). Primary CAFs are prone to senescence and loss of specific phenotypes following several generations of *in vitro* culture.^126^ To address this, plasmids (pBabe-SV40-T (*neo*)) have been used as vectors to transfect immortalization viruses into primary CAFs, establishing immortalized biochemical cells.^127^

#### Three-dimensional drug sensitivity testing model for CAFs-tumor cell interaction

Researchers have created a “3D slice model”, an advanced 3D cell culture model, to more accurately replicate the TME and tumor tissue architecture. The model co-cultures HNSCC cells and CAFs on a composite matrix of Matrigel and collagen I, creating a tissue-like 3D structure that precisely replicates the TME and tissue architecture (Figure 4). The model replicates the interaction between tumor cells and CAFs while providing adequate cell numbers for molecular investigation. In subsequent evaluations of drug sensitivity, researchers administered cisplatin to the 3D model and assessed cellular metabolic activity using WST-8 assays. At the molecular level, immunofluorescence was used to assess E-cadherin and vimentin expression and to evaluate the distribution and interactions between tumor cells and CAFs.^123^^,^^124^

#### Tumor cell and primary CAF co-culture spheroid model for animal tumor models and drug screening

Researchers have characterized tumors derived from 2D monolayers and 3D spheroids to produce highly standardized *in vivo* models. As a result, the main CAFs are isolated from HNSCC tissues. The CAFs are then co-injected with monolayer cells or spheroids into the oral mucosa to create heterotopic or orthotopic tumor models (Figure 4). Cisplatin injection significantly suppressed the growth of tumors produced from spheroids, in contrast to those originating from monolayer cells. The 3D spheroid model derived from HNSCC cells and primary CAFs can yield reliable results for chemotherapeutic drug screening.^123^

Zebrafish models have also been used to investigate HNSCC-related CAFs. One study examined the HNSCC metastatic cell line HSC-3 alongside five types of stromal cells: normal oral fibroblasts (NOFs), CAF, macrophages, CD4^+^ T cells, and human umbilical vein endothelial cells (HUVEC). HSC-3 cells were co-cultured with each stromal cell type and injected into zebrafish embryos at 2 days post-fertilization to model HNSCC tumors and assess the impact of stromal cells on tumor growth. CAFs were found to significantly promote tumor volume.^125^ When selecting experimental models, multiple techniques, including flow cytometry, immunofluorescence, and immunohistochemistry, should be employed to assess marker expression in specific CAF subpopulations. Monitoring should be conducted throughout the experiment to prevent marker loss, which could compromise the validity of the conclusions and hinder the reproduction of CAF dynamic behavior in advanced 3D organoid culture models.

Since CAFs vary by source, efforts should be made to minimize their potential interference with the study. First, a standardized CAF experimental system should be established. The patient origin of the CAFs, including the tumor site, TNM staging, differentiation grade, and prior treatment history, should be clearly defined.

Isolation methods, such as enzymatic digestion, tissue block extraction, and magnetic bead sorting, should be specified. Primary CAFs may exhibit variations. The criteria for the CAFs should be identified, including positive and negative markers and their respective positivity rates. The CAFs' passage number should be specified, and culture conditions should be defined, including the medium composition and the matrix gel type. A standardized system facilitates subsequent cross-study integration and analysis. Second, the CAF function at the subtype level should be interpreted. Studies should avoid generalizing conclusions from a single subtype to all CAFs and should use single-cell or spatial transcriptomics technologies to subtype CAFs and determine their relationship to chemotherapy resistance. Furthermore, key conclusions should be cross-validated across multiple models. To enhance reliability, mechanistic conclusions should be validated in ≥2 distinct models. Additionally, multicenter collaborative studies should be conducted. Results from single-center studies with a small number of patient-derived CAFs may be subject to sampling bias. Multicenter collaborations should be established to collect enough CAF samples. Methods such as meta-analysis should be used to identify core pro-resistance features that are consistent across studies and samples. Concurrently, an open-access functional database of CAFs should be created. This database should aggregate transcriptomic, proteomic, and functional data from CAFs derived from different research centers and patient sources. This will facilitate data integration and comparative analysis by researchers and accelerate the establishment of a consensus-based, unified conceptual framework in this field.

However, the single-cell technologies lack spatial context, hindering our understanding of how the local signaling microenvironment of CAFs influences their function.^128^ The reliability and accuracy of existing biomarkers in distinguishing the functional subgroups of CAFs are limited. The molecular characteristics, functional classification, and associations with clinical outcomes and treatment responses of different CAF subtypes remain to be fully elucidated, complicating the development of CAF-targeted strategies. The CAF phenotype is dynamically regulated by complex microenvironmental signals, such as cytokines, mechanical stress, and metabolites.^129^ CAF characteristics are easily lost *in vitro,* and animal models cannot accurately replicate the complexity of the TME.^130^ Most current research relies on *in vitro* cultures or single animal models. Although 3D models more accurately simulate the TME and tissue structure, they significantly differ from actual conditions.^123^^,^^124^ To address this limitation, more advanced 3D models that more closely replicate CAF dynamics have been used in research on CAF-mediated drug resistance. Patient-matched allogeneic organoid-CAF co-culture systems, for example, simultaneously isolate tumor cells and CAFs from the same patient specimen while preserving patient-specific tumor-stroma interactions. These systems can elucidate personalized cytokine resistance profiles in pancreatic cancer (abstract, Lee et al., Cancer Res 85, B050). Highly biomimetic solid tumor-CAF-immune triple-cell integration models have been used to evaluate the inhibitory effects of physical and chemical barriers on CAR-T cell efficacy.^131^ Additionally, while the technical platform using two-photon polymerization (2 PP) to create PEGDA microscaffolds for 3D cell culture has not yet been applied to cancer research, its superior biocompatibility and 3D microcage arrays, which provide precise physical boundaries and supportive scaffolds for cells, enable more accurate simulation of spatial interactions between CAFs and tumor cells.^132^

## Current status of targeting CAFS to enhance cisplatin sensitivity IN HNSCC

### Potential compounds targeting CAFs to enhance cisplatin sensitivity in HNSCC

To develop novel strategies to hinder the progression of HNSCC tumors, researchers have discovered compounds that can suppress the phenotypic characteristics of CAFs that promote resistance, therefore increasing cisplatin sensitivity in HNSCC. Nevertheless, the functions of these medicines remain poorly understood.^86^^,^^87^^,^^107^

CAFs derived from patients with primary cancer exhibit higher baseline autophagy levels than normal fibroblasts from patients with non-cancer. The administration of low-dose SAR405 (1.0 μmol/L) may diminish CAF autophagy. Furthermore, when combined with cisplatin, it demonstrates significant therapeutic efficacy in clinical practice.^107^ The IDO1 inhibitor navoximod was delivered with cisplatin in a study involving IDO1^+^ CAFs. The authors found that the combination significantly increased the frequency of DNA double-strand breaks (DSBs) and reduced chromosomal instability (CIN), thereby overcoming cisplatin resistance. Data from animal models demonstrated that the combination of navoximod and cisplatin significantly suppressed tumor proliferation and diminished tumor mass.^86^^,^^87^ Cetuximab, an anti-EGFR drug, effectively restores chemotherapy sensitivity in tumors composed of cancer cells and CAFs that are resistant to chemotherapy. This was disclosed through a study on chemotherapy-resistant CAFs.^85^

Crenigacestat significantly increased the sensitivity of cisplatin-resistant HNSCC cell lines in a CAF-HNSCC co-culture system, as evidenced by a prior *in vitro* study.^123^

Certain chemicals can enhance cisplatin sensitivity through interacting with CAFs. A novel rFab' anti-PD-L1 therapeutic was developed alongside immune checkpoint inhibitors (ICIs). ICIs not only impede the progression of HNSCC tumors via CAFs but also enhance the effectiveness of cisplatin treatment.^133^ Chen et al. discovered that combining an oncolytic virus (OV) with cisplatin treatment markedly reduced tumor spheroid development in their investigation of natural tumor products. Moreover, the extracellular vesicles containing mTOR, PI3K, STAT3, β-catenin, and miR-21-5p produced from CAFs were reduced.^102^

### A CAF-targeted delivery system improves cisplatin penetration and enhances treatment efficacy

CAFs secrete collagen, forming a dense mechanical barrier that impedes the penetration of chemotherapeutic agents into deeper tumor regions, thereby affecting treatment efficacy. To address this, some studies have sought to develop precision delivery systems targeting CAFs. These systems can block CAFs' pro-tumor functions and improve the TME. This enhances the efficacy of combination therapies and holds significant clinical importance.

CAF-targeting drug delivery systems significantly improve the penetration efficiency of cisplatin in tumor tissues, enhancing anti-tumor efficacy. One study developed a three-tiered delivery system consisting of micelles, liposomes, and a thermosensitive gel, co-loaded with a cisplatin (IV) prodrug, losartan, and a PD-1 antibody. In this system, losartan reprograms CAFs by inhibiting the TSP-1/TGF-β1/Smad2/3 pathway, thereby reducing ECM deposition and significantly enhancing platinum drug accumulation and deep penetration. Drug accumulation analysis showed that the intraperitoneal-to-plasma area under the curve (AUC) ratio at the tumor site was 3.6 times higher than that of the free drug, demonstrating good treatment safety.^134^ Another study utilized fibroblast membrane-mimicking liposomes co-loaded with a cisplatin prodrug and ginsenoside PPD. Downregulating the p38 MAPK pathway with this approach inhibited cisplatin-induced CAF senescence and SASP formation. This reversed the suppressive effects of CAFs on the tumor immune microenvironment. This resulted in a 2.2-fold increase in tumor-site accumulation while avoiding the hepatotoxicity associated with free cisplatin.^135^

Other studies have explored drug delivery system strategies that target CAFs, focusing on reducing the matrix to break down physical barriers and open pathways for chemotherapy drugs to penetrate tumor tissue. Some studies have used nanocarriers to deliver losartan,^136^ SIS3,^137^ navitoclax,^138^^,^^139^ tranexamic acid,^140^ quercetin,^141^ celastrol,^142^ or α-amyrin^143^ to induce apoptosis or inactivate CAFs and reduce ECM production. Furthermore, targeting the Golgi apparatus of CAFs with all-trans retinoic acid inhibits the synthesis and secretion of collagen and fibronectin, thereby suppressing ECM production at its source.^144^

Compared with free drugs and non-targeted chemotherapy sensitizers, CAF-targeting delivery strategies have better safety profiles. Because the drugs are more concentrated in tumor tissue, their distribution to major toxic target organs, such as the heart, liver, and kidneys, is significantly reduced. Nanoencapsulation can alleviate the cardiotoxicity of doxorubicin,^138^ the hepatotoxicity of celastrol,^142^ and the thrombocytopenia associated with navitoclax.^139^ Additionally, the carrier materials used in targeted delivery systems, such as carbon dots, albumin, modified starch, phospholipids, and red blood cell (RBC) membranes, have favorable biodegradability and clinical-use profiles, with no evidence of significant accumulation, toxicity, or severe immunogenic reactions observed *in vivo*.^136^^,^^143^^,^^144^^,^^145^

CAF-targeted delivery systems have demonstrated significant potential in preclinical studies; however, numerous challenges remain for clinical translation. The heterogeneity of the TME can lead to variability in the efficacy of responsive nanomedicines among patients. This necessitates selecting patients based on biomarkers, such as FAP-α expression levels and baseline IFP values.^146^ Sequential-release systems also require highly precise formulation processes and quality control, and maintaining batch-to-batch consistency remains a major challenge for industrialization.^141^

Currently, completed clinical trial results directly validating the efficacy of therapies targeting CAFs in HNSCC are lacking. Strategies aimed at eliminating CAFs in HNSCC remain controversial. In HNSCC, FAP^+^CAFs neither promote tumor growth nor enhance resistance to PD-1 inhibitors, challenging the feasibility and efficacy of targeting FAP.^147^ Depleting CAFs may enhance tumor invasiveness and metastasis.^148^ Certain CAF subpopulations, such as quiescent or “tumor-suppressive” CAFs, may play a “restraining” role by maintaining matrix structural integrity, thereby limiting tumor cell spread and regulating immune surveillance.^149^ However, excessive CAF clearance and ECM degradation can lead to a loss of structural support for intratumoral blood vessels, resulting in vascular collapse or irregular dilation. This exacerbates intratumoral blood flow heterogeneity and hypoxic conditions, further activating signaling pathways, such as HIF-1α, and inducing tumor cell resistance to chemotherapy.^150^^,^^151^^,^^152^ Certain CAF subpopulations, such as iCAFs and apCAFs, are involved in regulating immune responses; indiscriminately clearing these populations is likely to reduce their anti-tumor effects.^81^^,^^82^ Given the limitations in basic CAF research, such as an incomplete classification system and unclear specific markers, existing strategies for targeted CAF clearance largely rely on a few markers, such as FAP-α and α-SMA.^153^ However, these markers are not unique to CAFs. For example, α-SMA is widely expressed in renal stromal cells; targeting it may induce nephrotoxicity.^153^ In summary, the lack of truly CAF-specific targets hinders the development of precise elimination strategies and carries a significant risk of off-target toxicity.

Consequently, research on strategies that target CAFs has shifted from “targeted elimination of CAFs” to “targeted reprogramming of CAFs”. The latter focuses on modulating the functional state of CAFs to transform “tumor-promoting” CAFs into “tumor-suppressing” CAFs or induce a quiescent state rather than killing them. This approach interferes with the stroma’s supportive role for the tumor while preserving the integrity of the stromal structure. Thus, the stroma’s natural restraining function on the tumor is restored. A study of triple-negative breast cancer demonstrated that targeting CAF reprogramming yielded objective clinical efficacy. The Phase I EDALINE clinical trial combined sonidegib, an SMO inhibitor, with docetaxel chemotherapy to target the Hedgehog signaling pathway and reprogram CAFs. Among 12 patients with metastatic disease, 3 achieved clinical benefit, including 1 with complete remission. These results demonstrate that reprogramming CAFs via the stromal remodeling pathway can reverse chemotherapy resistance and produce clear therapeutic effects.^154^ These findings provide proof of concept for the combined strategy of “stromal modulation + chemotherapy”.

### The potential value of CAF markers associated with cisplatin resistance in diagnostic and patient stratification strategies

To date, data on cisplatin resistance in HNSCC remain lacking; however, findings from basic research offer insights for clinical strategies. For example, microarray analysis has suggested a link between high matrix metalloproteinase 7 (MMP-7) expression and cisplatin resistance in HNSCC, suggesting MMP-7 as a biomarker for predicting cisplatin response.^155^ Pan-cancer studies have revealed additional mechanisms by which CAFs mediate cisplatin resistance. For example, in lung squamous cell carcinoma, IL-6 activates the PI3K/AKT/NF-κB signaling axis, which in turn activates SP1 and the drug efflux protein ABCB7, attenuating the cytotoxic effects of cisplatin and paclitaxel. Furthermore, elevated serum IL-6 levels may serve as a liquid biopsy biomarker for predicting poor chemotherapy response.^156^ In gastric cancer, immunohistochemical results showed that in the group resistant to 5-fluorouracil (5-FU) combined with cisplatin chemotherapy, the positive expression rate of PPIC, which encodes pro-cytotoxin C, was 69.7%, significantly higher than the 43.6% observed in the sensitive group.^93^

These findings provide a theoretical basis for patient stratification strategies based on CAFs’ functional heterogeneity. Serum IL-6 or IL-8 levels and ^68^Ga-FAPI PET/CT imaging parameters can predict patient responses to cisplatin and guide personalized treatment regimen development.^156^^,^^157^ However, these approaches require validation in prospective clinical trials. In gastric cancer, the PPIC-labeled subpopulation of CAFs is associated with chemotherapy resistance. This subpopulation can be used to identify patients who may be resistant to cisplatin or 5-FU neoadjuvant chemotherapy and guide the selection of alternative treatment regimens.^93^

Research on CAF markers associated with cisplatin resistance remains limited, and the universality of existing biomarkers across different tumor types has yet to be validated. Some marker molecules, such as FAP, are widely expressed in normal tissues and lack sufficient specificity. Most studies have focused on correlation analysis, and causal evidence remains insufficient. Clinical validation is often based on retrospective samples and lacks support from prospective cohort studies. Additionally, CAF heterogeneity hinders a single biomarker from reflecting all resistance mechanisms.

Future efforts should leverage technologies such as single-cell sequencing and spatial transcriptomics to characterize functional subpopulations of CAFs. Additionally, prospective clinical studies are warranted to advance the translation of biomarkers into clinical practice. Overall, the high heterogeneity of CAFs poses a major challenge to precise stratification. Future stratification strategies should focus on specific CAF subtypes rather than on overall CAF burden.

## Challenges and perspectives

Cisplatin resistance remains a major challenge in the clinical management of HNSCC. Previous studies have primarily focused on tumor-intrinsic mechanisms while overlooking other components of the TME. Investigating how CAFs regulate cisplatin responsiveness in HNSCC may thus provide novel therapeutic perspectives. Although this research area is less developed than comparable work in ovarian^158^ and colorectal cancers,^118^ it offers important opportunities for advancing HNSCC treatment.

However, research on CAF-associated cisplatin resistance in HNSCC has not been translated into clinical application. In cancer therapy, resistance to chemotherapeutic agents such as paclitaxel,^55^^,^^56^ and EGFR inhibitors^159^^,^^160^ has long been associated with CAFs. Clinical studies of HNSCC have shown that early cetuximab treatment can induce EMT and CAF activation,^161^ but comparable clinical evidence for cisplatin remains limited. For example, high CAF autophagy in HNSCC promotes cisplatin resistance,^105^^,^^106^^,^^107^ and autophagy inhibitors significantly enhance cisplatin efficacy in mouse models.^107^ Nevertheless, further clinical research is needed, and current evidence remains insufficient to fully evaluate the cisplatin-sensitizing effects of autophagy inhibitors. In pan-cancer studies, early clinical trials targeting CAFs have generally failed.^162^^,^^163^ Olaratumab, an antibody targeting PDGFRα, ultimately failed to improve outcomes,^162^ while a phase II trial of the HGF-targeting agent ficlatuzumab showed only a 19% objective response rate in patients with cetuximab-resistant HNSCC.^163^

These clinical findings suggest that targeting CAFs alone lacks sufficient specificity to distinguish among functionally distinct CAF subtypes, underscoring the challenge posed by CAF heterogeneity. More selective inhibitors against markers of specific CAF subpopulations are therefore needed. Inhibiting PDGFRα alone appears insufficient to delay tumor progression, contradicting several preclinical observations.^162^^,^^164^^,^^165^ This discrepancy suggests that current basic CAF research often describes what CAFs are but does not adequately explain why they acquire specific functional states. A similar limitation exists in studies of CAF-mediated cisplatin resistance in HNSCC. Many studies show that CAFs alter tumor-cell sensitivity to cisplatin through paracrine signaling, but the key molecules involved remain unclear. This uncertainty limits targeted drug development and reduces the translational value of preclinical findings. Mechanistic studies directly linking CAFs to cisplatin resistance are also rare in pan-cancer research. Only two studies have established direct links between CAFs and cisplatin resistance mechanisms. One study showed that CAF-secreted CXCL14 binds CCR7 on bladder cancer cells, activates STAT3 phosphorylation, upregulates *ERCC4*, and enhances cisplatin-induced DNA damage repair.^166^ Another study found that CAFs increase intracellular glutathione levels in tumor cells, promoting cisplatin efflux and resistance.^117^

Research on the immune microenvironment in CAF-mediated cisplatin resistance in HNSCC also remains limited. Although early preclinical studies indicate that CAFs can modulate the immune microenvironment, and numerous clinical trials have evaluated combinations of cisplatin and PD-1 inhibitors, detailed mechanistic investigations connecting these areas are scarce. One study showed that cisplatin-resistant IDO1^+^ CAFs promote an immunosuppressive microenvironment in HNSCC. Similarly, preclinical studies suggest that IDO1 inhibitors may enhance cisplatin sensitivity and immunotherapy efficacy,^86^^,^^87^ but subsequent clinical trials failed to demonstrate clear clinical benefit.^26^ IDO1 inhibitors have several limitations, including incomplete blockade of the kynurenine pathway and potential stabilization of non-enzymatic IDO1 conformations that may promote tumor progression.^167^ These findings suggest that conventional catalytic inhibition may be insufficient to fully reverse IDO1-mediated immunosuppression.

Accordingly, next-generation strategies, including PROTAC-mediated degradation and inhibitors targeting apo-IDO1, are being actively explored.^168^ Among pan-cancer studies, only one report has clarified how CAF metabolic reprogramming interacts with CD8^+^ T cells to overcome cisplatin resistance. They found that CAFs promote resistance by releasing glutathione and cysteine, increasing intracellular GSH levels in tumor cells, chelating cisplatin, promoting drug efflux, and reducing nuclear platinum accumulation and DNA damage. CD8^+^ T cell-derived interferon-γ (IFN-γ) reprograms CAF metabolism through the JAK/STAT1 pathway, reducing GSH and cysteine release, restoring nuclear cisplatin accumulation and DNA damage, and resensitizing resistant cells to cisplatin.^117^ This work supports combined immunometabolic therapy as a potential strategy for overcoming resistance and provides insights for developing new strategies for cisplatin-resistant HNSCC.

HNSCC arises across anatomically complex sites, including the oral cavity, pharynx, and larynx, contributing to substantial heterogeneity in the TME. CAF subtypes differ markedly in collagen deposition, inflammatory factor secretion, and treatment responses according to anatomical location.^169^^,^^170^ Dynamic microenvironmental changes can further drive CAF differentiation into distinct subtypes. In pan-cancer studies, cisplatin-resistant tumor cells can reactivate normal fibroblasts and transform them into CAFs by secreting lactate,^166^ exosomal PKM2,^171^ and other metabolic reprogramming factors, thereby forming a positive feedback loop that amplifies resistance. For instance, in bladder cancer, CAF-secreted CXCL14 enhances tumor-cell DNA repair, while lactate derived from tumor-cell glycolysis activates CAFs and upregulates CXCL14 expression. This tumor-CAF co-evolution involves metabolic reprogramming, epigenetic regulation, and extracellular vesicle communication, but existing co-culture models poorly capture these dynamic processes, resulting in fragmented mechanistic insights.^166^ Phenotypic plasticity among CAF subpopulations also complicates research, as CAFs can shift states in response to signals such as TGF-β and IL-1.^172^ Drug exposure may further reshape CAF phenotypes across cancers. For instance, cisplatin-induced CCL5 secretion by CAFs in ovarian cancer may enhance cisplatin resistance.^173^ Moreover, preclinical models often fail to recapitulate authentic tumor-stroma interactions *in vitro*, leading to loss of specific CAF phenotypes and limiting mechanistic investigation.

Future research should examine how dynamic temporal and spatial changes in the TME shape CAF behavior. Rather than focusing narrowly on individual CAF subtypes, studies should characterize CAF differentiation trajectories and the interactions among CAFs, tumor cells, immune cells, ECM components, and metabolic signals. Overlooking this broader microenvironmental crosstalk may distort mechanistic conclusions and hinder clinical translation. Experimental model development should prioritize stable organoid and animal models that accurately reproduce TME dynamics, moving beyond simplified 3D systems to more precisely identify CAF phenotypes associated with cisplatin resistance. This approach would allow researchers to move beyond simplistic 3D models and more precisely identify CAF phenotypes associated with cisplatin resistance. In clinical studies, HNSCC patient enrollment should account for tumor site, stage, prior treatment history, HPV status, and sex, rather than grouping all cisplatin-treated patients. The efficacy of combining targeted delivery systems with cisplatin and other chemotherapeutic agents should also be systematically evaluated.

## Acknowledgments

We are extremely grateful to the professors for fostering a relaxed and open research environment, as well as for their thorough guidance throughout the manuscript’s development. We would be remiss if we did not also acknowledge the contributions of the other authors for their diligent collection and organization of relevant research materials, which collectively enabled the completion of this manuscript.

This work was supported by grants from the Zhejiang Provincial Program for the Cultivation of New Heath Talents to Yiwen Zhang; 10.13039/501100005199Chinese Medicine Research Program of Zhejiang Province (grant nos 2022ZB017); and the Medical and Health Research Program of Zhejiang Province (grant nos 2022KY069).

## Author contributions

Conceptualization, D.L., D.Y., M.C., T.D., and Y.Z.; writing – original draft, D.L.; writing – review and editing, D.L., J.Z., and Y.Z.; funding acquisition, Y.Z.; visualization, D.L.; supervision, D.Y., X.L., D.S., and J.Z.; project administration, X.L.

## Declaration of interests

The authors declare that they have no known competing financial interests or personal relationships that could have appeared to influence the work reported in this paper.
